# Ecological Civilization and High-Quality Development: Do Tourism Industry and Technological Progress Affect Ecological Economy Development?

**DOI:** 10.3390/ijerph20010783

**Published:** 2022-12-31

**Authors:** Wei Yang, Qiuxia Chen, Yanyue Dao, Xiaoting Huang, Weifang Shao

**Affiliations:** 1 School of Management, Shandong University, Jinan 250100, China; 2 Yellow River National Strategic Research Institute, Shandong University, Jinan 250100, China

**Keywords:** tourism industry, technological progress, ecological economy development, PVAR model, entropy method, environmental economics, impulse response, ecological economics, sustainable development

## Abstract

The tourism industry is considered a smokeless industry or green economy. Under the circumstances of carbon peaking and carbon neutrality, it is essential and urgent to explore whether the tourism industry and technological progress can promote ecological economy development. Based on the panel data of 30 provinces in mainland China from 2007–2019, this paper, for the first time, incorporates the tourism industry, technological progress, and ecological economy development into the analytical framework by constructing a PVAR model. In addition, this paper calculates the indicator weights of each variable using the entropy weighting method. This paper utilizes GMM tests, impulse response analysis, Monte Carlo simulation, and variance decomposition to empirically investigate the dynamic impact mechanism of variables interacting with each other. The conclusions are as follows. First, the tourism industry always contributes positively to ecological economy development, while technological progress can facilitate ecological economy development in the long run rather than in the short term. Second, the tourism industry also positively contributes to technological progress. Third, ecological economy development has a “crowding out effect” on the tourism industry. Fourth, the tourism industry in developed eastern regions has a more powerful impact on ecological economy development than in underdeveloped middle and western regions. Based on the empirical results, we provide practical implications: first, the assessment system of the regional economy should include ecological development indicators; second, the tourism industry should accelerate the use of clean energy and the transformation of green technological innovation.

## 1. Introduction

Globalization and neoliberalism have had a huge impact on the contemporary world. It has increased the frequency of exchanges, flow between factors, and promoted technological progress in regions [1,2]. It has ushered in a new era of economic prosperity, opens up broad avenues of development, and pushes people into leaving homes and going on a trip [3,4,5]. However, globalization and neoliberalism have also raised a number of issues, the most prominent of which is the impact on the environment [6]. With the gradual worsening of global environmental problems and the consecutive spreading of the pandemic, all countries meditate on concepts that can lead and coordinate development to ensure economic recovery, promote ecological economy development, and satisfy people’s needs for living a happier life [7]. Since the launch of reform and opening up, China’s economy has achieved leapfrog development [8] while paying a huge ecological and environmental cost [9,10]. It is noteworthy that China still faces prominent problems such as excessive income inequality, unbalanced development, and ecological and environmental problems [8,11,12]. Ecological economy development is a brand-new development concept proposed by China, including innovation, coordination, green, openness, and sharing. Ecological economy development is the efficient “conjugation” of various elements in the development process and results of the economic system [13], which is an inevitable option for China to start a new development stage, implement the new development concept, and build a new development pattern. Technological progress is the pivotal strategic support for building a modernized economic system. In addition, the 14th Five-Year Plan further emphasizes the leading role of technological progress and innovation of science and technology in promoting ecological economy development.

The latest Chinese Central Government report points out that Chinese modernization is a harmonious coexistence between humankind and nature and indicates respecting, adapting to, and protecting nature, which is an inherent requirement for comprehensively building a modern socialist country. To embark on the new journey of building a modern socialist country in an all-around way, government departments must firmly implement the concept that clear waters and green mountains are as good as mountains of gold and silver, and pursue development based on harmonious coexistence between humankind and nature. The government will further resolutely give up the traditional mode of national modernization that disdains, dominates, and destroys nature, and transform the traditional mode into the new one that respects, adapts to, and protects nature. We will unswervingly follow the path of ecological priority and green development, and construct modernization featuring harmonious coexistence between humankind and nature. Under the premise of protecting nature, we should establish an ecological economic system with industrial ecology and ecological industrialization as the main body. Moreover, we should try our best to achieve financial value of waters and mountains while keeping waters clear and mountains green. China will strive to achieve peak carbon dioxide emissions by 2030 and carbon neutrality by 2060 [14]. Chinese modernization is a modernization that embodies green and sustainable development and integrates ecological development into overall development.

As the policy of reform and opening up has been active for more than 40 years, the tourism industry has become an essential part of China’s national economy. The tourism industry has been considered a “smokeless industry”, which makes full use of modern science and technology to activate new dynamics of industrial development and start a new pattern of industrial development. It is an organically integral part of promoting ecological economy development. The development of the tourism industry has become an important way to improve people’s lives, enhance their well-being, and satisfy their needs for a better life [15]. Meanwhile, the tourism industry development highlights harmony and unity with the economy, society, environment, and resources. Moreover, the tourism industry covers multiple industries and boosts the development of urban and rural areas. The development of tourism resources also depends on the coordinated development of ecological protection and the environment. Promoting the continuous development of the tourism industry is one of the important ways to build an ecological civilization society and move towards Chinese modernization. At present, China’s tourism industry is entering a new stage of quality and efficiency improvement from the stage of brutal growth. Therefore, exploring the interactive influence relationship among the tourism industry, technological progress, and ecological economy development can not only find new momentum for the tourism industry but also find evidence for the driving and spillover effects of technological progress on the tourism industry and national economy. In view of this, research questions are proposed: (1) Do the tourism industry and technological progress affect ecological economy development? (2) How do the tourism industry, technological progress, and ecological economic development interact with each other?

Firstly, based on the panel data of 30 Chinese provinces from 2007 to 2019, the entropy method is used to accurately measure the tourism industry, technological progress, and ecological economic development. Secondly, this paper is the first to incorporate the tourism industry, technological progress, and ecological economy development into the analytical framework and empirically explores the dynamic relationship of their mutual influence through the panel vector autoregression model, and we obtain several pivotal conclusions. Finally, this study uses panel data for heterogeneity analysis.

## 2. Literature Review

### 2.1. The Tourism Industry and Technological Progress

The tourism industry integrates the primary, secondary, and tertiary industries, covering agritainment, manufacturing cultural and creative souvenirs, offering tourism equipment and entertainment facilities, as well as scenic cable cars, car ferries, and other transportation vehicles. The tourism industry provides rich landing and application scenarios for technological progress, involving clothing, food, housing, and transportation. For example, the penetration of digital technology advancement in the tourism industry is empowering this industry, which can not only improve the efficiency of the tourism industry and improve the tourism experience comprehensively but also transform the tourism development mode and inject new vitality and new momentum for the ecological economy development of the tourism industry [16]. In November 2020, the Ministry of Culture and Tourism and ten other departments issued the “Opinions on deepening ‘Internet + Tourism’ to promote ecological economy development of the tourism industry”, proposing that the tourism industry should adopt technological empowerment to achieve further transformation of the quality, efficiency, and dynamic of the tourism industry. In December 2021, the State Council launched the “14th Five-Year Plan” to promote the sustainable and healthy development of the tourism industry, to better play the role of the tourism industry in promoting ecological economy development, and to meet people’s needs for a better life. The plan also proposed to enhance the tourism industry development with technological progress. Technologies can be used to help the tourism industry patterns, supply modes, business models, and consumption patterns transform, such as in big data, cloud computing, the Internet of Things, blockchain, 5G, the Beidou Navigation Satellite System, virtual reality, augmented reality, and other applications. There will be a new momentum that can propel the tourism industry development and help construct the new economic development pattern of the large domestic cycle as the mainstay and domestic and international dual-cycle development patterns reinforcing each other [17]. Furthermore, the tourism industry has a strong industrial linkage and is gradually becoming a bridge between the secondary and tertiary industrial clusters. The tourism industry pulls the production and manufacturing sectors of the secondary industry and the leisure products, shopping, and services sectors of the tertiary industry. In addition, the pulling power of industry linkage is particularly evident and catalyzes technological progress by linking these sectors [18]. Romão et al. (2019) analyzed whether regional technological progress affects the tourism industry development in Europe and how by using the spatial econometric method to study 237 European regions [19]. Pan et al. (2021) calculated the coupling coordination degree of China’s tourism industry and economic development with the data envelopment analysis (SBM-DEA) model based on the statistical data of 30 provinces in China from 2007 to 2017 [20]. Gössling et al. (2019) explored the idea that the continuous advancement of information and communication technology laid an elementary foundation for the emergence of the new P2P business model for the shared accommodation industry [21]. The shared accommodation industry has revitalized many idle resources in tourist destinations and promotes the consumption and sustainable development of the tourism industry. Technological progress can drive the tourism industry and bring new business models and product formats that can promote tourism consumption and, thus, tourism economic development. That is to say, there is a good interactive influence relationship between the tourism industry and technological progress [22]. For one thing, compared to traditional technological progress, technological progress related to the tourism industry attaches more importance to the technological dispersion of environmentally friendly, clean production, resource-saving, and sustainable use technologies, aiming to develop toward the direction of technological empowerment and sustainability [23,24]. For another, technological progress will facilitate the tourism industry in the long-term, but in reality, the impact of technological progress on the tourism industry development in different regions and time periods is characterized by inconsistency and lack of synchronization due to the high cost of technological progress, the long period, and the difficulty of transforming the fruits [25,26].

### 2.2. Tourism Industry and Ecological Economy Development

The tourism industry plays a prominent role in improving the regional infrastructure and regional ecological environment. The tourism industry has become one of the strategic pillar industries in China, and research about the impact of the tourism industry and ecological economy development is quite fruitful. Adedoyin et al. (2021) studied the impact of the tourism industry on economic growth from the perspective of institutional quality [27]. Danish et al. (2018) studied the relationship between the tourism industry and economic growth from a two-way dynamic interaction [28]. Liu et al. (2017) explored the role of the tourism industry in influencing economic development from the perspective of economic structure [29]. Ivanov et al. (2013) analyzed the impact of the tourism industry on economic development in Asia, America, and other countries through growth decomposition methods [30]. Lin, V.S. et al. (2019) used the Bayesian probit model to study the interrelationship between outbound tourism and regional economic development in 29 countries and regions [31]. Stauvermann et al. (2016) theoretically analyzed the impact of changes in the foreign currency revenue of tourist-generating countries on the economic growth of small tourism-dependent islands [32]. Paramati et al. (2017) empirically investigated the dynamic relationship between the tourism industry and economic growth by using robust panel econometric techniques and compared the impact of the tourism industry on economic growth in developed and developing economies [33]. The results showed that the tourism industry has a significantly positive impact on economic growth in both developed and developing economies, supporting the general hypothesis that tourism flourishes in economic growth. Other scholars have also explored the two from different perspectives. Various methods have been used to study the inbound tourists’ consumption in the host country as a way to boost the local economy [34], such as the propensity score matching technique [35], the Panel Vector Auto-Regressive (PVAR) model [36], the cross-sectional data analysis [37], the panel data unit root test and co-integration analysis [38], spatial econometric analysis based on new economic geography [39], fuzzy set qualitative comparative analysis (fsQCA) [40], social nucleic acid matrix analysis [41], and other methods [28,42]. The tourism industry may be the engine in rural areas where there is a relative lack of capital, technology, infrastructure, and human resources to support large-scale industrial development that drives the local economy. Currently, China is still a developing country with a vast amount of rural areas. The tourism industry can promote the economic development of rural areas and facilitate the improvement and upgrading of basic infrastructure [43,44]. The tourism industry is able to boost economic development by balancing international payments [45], attracting overseas investment [46], increasing tax revenue [47], creating jobs [48], and increasing the demand for local products and services [49,50,51,52].

### 2.3. Technological Progress and Ecological Economy Development

The impact of technological progress on economic development originated from Schumpeter’s theory of technological progress driving economic development. The new growth theory and the endogenous growth theory consider technological progress as an endogenous driver of economic development and a decisive driver of efficiency enhancement, on which academics have reached a consensus [53]. For example, Rome (1986) argues that the spillover effect of technological progress due to knowledge and technology becomes a necessary condition for economic development and that technological progress is the main cause of regional economic development [54]. Pradhan et al. (2022) concluded that technological progress significantly contributed to the economic development of Europe [55]. Maradana et al. (2017) found that the relationship between technological progress and economic growth has both unidirectional and bidirectional causality [56]. Zhou et al. (2021) used a nonlinear econometric model with provincial data from 2000–2014 to investigate the impact of technological progress and structural change on economic growth in China [57]. From a national perspective, there is an inverted U relationship between technological progress and economic growth, suggesting that the mode of technological progress needs to shift from imitation to innovation. When the turning point occurs, structural upgrading will stimulate China’s economic growth. Liu et al. (2021) used data from 278 prefecture-level cities in China from 2003–2017 and used a data envelopment analysis game cross-efficiency model to explore the relationship and mechanisms between technological progress and economic efficiency [58]. Du et al. (2021) argued that technological innovation and industrial structure upgrading can promote economic transformation [59], while Lucas (1988) argues that technological progress caused by human capital spillover is the source of economic growth [60]. Scholars have conducted a large number of empirical studies on the relationship between technological progress and economic development at several levels and obtain very different conclusions. They can be summarized as follows. Firstly, technological progress can significantly improve regional total factor productivity, promote regional economic development, and cultivate competitive advantage [61,62]. Secondly, in the short term, technological progress will exist in the form of costs and having a “crowding out effect” of resources on the core business of the enterprise, which, in turn, will lead to the contradiction between the economic efficiency of the enterprise and the overall economic development [63]. Next, the impact of technological progress on economic development is affected by various factors, thus showing different indirect impact effects [64,65]. Finally, technological progress not only gives rise to the digital transformation of enterprises, upgrading of government digital governance, and further catalyzing the vitality of market digitalization, but also induces new economic business models and new forms of consumption so as to contribute to ecological economy development [66,67,68].

### 2.4. Literature Commentary and Marginal Contribution

The research results of the existing literature need to be further complemented; first, the existing studies focus mainly on the static relationship between tourism development and environmental quality, while ignoring the dynamic influence processes of the tourism system. However, since the entire system constituted by the tourism industry is dynamic, the elements in it are highly mobile [69]. Second, the existing literature is more traditional in the selection of variables for economic development, which results in a lack of selection of ecological indicators and short of identification of ecological attributes [70,71]. Third, the existing literature does not place the tourism industry, technological progress, and ecological economic development in the same framework; especially, the lack of research results on technological progress is an important explanatory variable among the three [28,72]. Considering the “Porter hypothesis”, this may seriously neglect the effect of technological progress on ecological development. Therefore, it is urgent to explore and improve the impact and interaction mechanism of the tourism industry and technological progress on ecological economic development, which is the focus of this study.

Based on the literature review and theoretical analysis, it is obvious that existing studies have either focused on the relationship in pairs between the tourism industry, technological progress, and economic development, or they have taken technological progress as one of the elements to qualitatively explore its role and significance in the relationship between the tourism industry and ecological economy development. Therefore, this paper intends to improve and extend the analysis in four aspects. First of all, constructing a new analytical framework by means of a panel vector autoregression (PVAR) model, which places China’s tourism industry development, technological progress, and ecological economy development in the same analytical framework and considers them as endogenous variables. This method does not require research hypotheses and proposing independent and dependent variables ahead of time and can directly verify the interrelationships and action mechanisms through the model. It can control individual and temporal effects that are not easily observed due to spatial variations and help clearly characterize the transmission mechanisms of various shocks and observe the relative importance of random disturbance terms that affect the variables. After that, in this study, industrial base, industrial input, and industrial output, including nine types of indicators, are set in the tourism industry development indicators. The entropy method is used to calculate the weights, which further ensures the scientificity and accuracy of the indicators’ selection. Moreover, in terms of ecological economy development, since the economic system is a complex and grand system including multilevel elements, we take the background into consideration that China’s economic and social development has entered a new era, a new situation, a new stage, and a new goal. This study adopts the new development concept of “innovation, coordination, green, openness, and sharing” to guide the selection of indicators. Finally, we select 18 indicators at 5 levels, which depict the rich connotation of ecological economy development as comprehensively as possible. Furthermore, the Granger causality test and dynamic impulse response analysis were conducted to study the relationship among the tourism industry, technological progress, and ecological economy development as well as their mechanism from the bidirectional and dynamic perspectives so as to explicitly exhibit the short-term and long-term relationship trend and influence degree.

## 3. Theoretical Mechanism and Hypothesis Development

### 3.1. Environmental Economics and Ecological Economics

When it comes to environmental economics, the concept of “sustainable development” needs to be mentioned. Sustainable development was first proposed by the Club of Rome, and, so far, one of the most popular definitions is that sustainable development is the development that “meets the needs of the contemporary generation without jeopardizing the ability to satisfy the needs of future generations” [73,74]. Thus, the emphasis of sustainable development is still on development and growth, and the concept is applicable to many aspects, not merely confined to economics. Internalizing environmental values is an especially prominent theory in environmental economics. According to neoclassical economists, environmental problems are due to the inefficient use of natural resources and the undervaluation of natural resources [75]. For neoclassical economics, the environment has externalities. From the perspective of environmental economics, the reason why environmental problems conflict with economic development lies in externalities. Therefore, it is necessary to assess the value of the environment, convert it into a price, and at the same time identify the property rights of the environment [76,77,78]. One of the main assumptions of this view is that man-made and natural capital are replaceable [79]. That is to say, economic growth and sustainable use of natural resources can be achieved simultaneously.

This so-called “Porter hypothesis” deserves special attention because it assumes that there are win-win solutions for both economic development and the protection of the natural environment [80]. They propose that a series of measures such as environmental regulation will stimulate technological innovation and improve company performance, contributing not only to environmental protection but also to economic growth [81]. It is optimistic about the global ability to cope with a series of crises that may arise from resource depletion, environmental degradation, etc. [82].

Ecological economics is based on the theory that the cause of all environmental problems lies in the misallocation of capital [83]. It proposes several new ways to solve the problem on a basis of traditional approaches such as tax increases. It is eye-catching to advocate that we need to view the natural environment as capital and invest in it while improving the efficiency of resource use [84]. Under the circumstances of the economic depression caused by the globally rampant pandemic, it appeals to boosting economic growth through investment, which is the main reason to be concerned. It can also be said that the ecological economy is a new economic model based on the concept of weak sustainability [85,86].

China is still the largest developing country in the world, with a low per capita national economic level and highly uneven development between regions. If China wants to skip the “middle-income trap” [87], it must attach great importance to economic development and adopt a mode of governing, while developing in order to sustain the economic and social development of China. Under such circumstances, ecological economic development emerges as a new model of sustainable development that is different from the traditional one.

Ecological economic development is a new development concept for Chinese modernization. The new development concept contains innovation, coordination, greenness, openness, and sharing. It is a development direction and development goal that are based on the root, control the overall situation, and focus on the future. It is a macro strategy aiming at optimizing the economic structure, converting the development model, and enhancing the development momentum in order to cope with the complex internal and external environment and address the current development problems, based on a comprehensive judgment of the environment of the era and giving full play to the advantages of the institution. Ecological economy development can meet the growing needs of people seeking a better life and reflect the implementation of the new development concept. The concept depicts that innovation is the first driving force, coordination is an endogenous feature, green becomes the universal state, openness is the necessary path, and sharing becomes the fundamental purpose.

### 3.2. Mechanism Analysis and Hypothesis Development

Actually, ecological economy development means innovation and transformation of the growth mode. Technological progress drives ecological economy development through the “re-balancing mechanism of supply and demand”, containing two aspects of the driving mechanism. Firstly, technological progress drives the quality transformation of supply and demand structures, coordinates and optimizes the supply and demand structures, and promotes the improvement of supply and demand quality [64,65]. Secondly, the quality transformation and coordination of the internal structure of supply and demand will further drive the re-balancing and coordination between supply and demand, thus promoting economic coordination and allowing development to benefit more industries, so as to realize the goal of coordination and sharing of ecological economy development. What is more, technological progress will empower the ecological economy development of the tourism industry. With the full integration of digital technology into the tourism industry, the level of digitalization of the tourism industry continues to improve. Thus, tourism production, experience patterns, service ways, and governance methods begin to take on digital trends so that digital technology transforms the tourism industry in all aspects, from multiple angles, and through the whole chain to improve the efficiency of the tourism industry [63]. It also promotes the level of government management. The intelligentization of the product, personalized satisfaction of consumer demand, online business services, and other new business models [66,67] can improve the quality and efficiency of tourism enterprises’ products and services and stimulate the new vitality of the tourism industry [68]. In terms of spatial dimension, the acceleration of industrial factor flow and the improvement of industrial efficiency have a significant spillover effect on the neighboring provinces [88].

**Figure 1:**
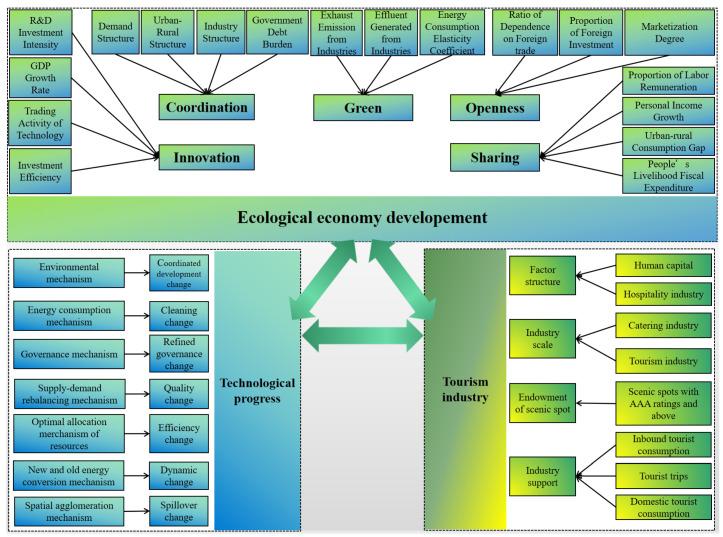
Influencing mechanism framework of the tourism industry, technological progress, and ecological economy development (source: made by authors).

To sum up, the tourism industry is one of the pivotal bridges connecting the secondary industry cluster and the tertiary industry cluster within the country and has a strong radiation effect on multiple industrial sectors [16]. With the increase of modern tourists and the further upgrading of tourism consumption, the driving capacity of this industry becomes very prominent [17], leading to technological progress in many industry sectors [18]. Therefore, we propose our hypotheses: (1) the tourism industry and technological progress significantly positively affect ecological economy development; and (2) the tourism industry, technological progress, and ecological economic development interact with each other. In view of the above analysis, this study sorts out the relationship between the tourism industry, technological progress, and ecological economy development, proposing the following theoretical framework (Figure 1).

## 4. Research Method, Sample Selection, and Data Sources

### 4.1. Research Method and Research Scheme

#### 4.1.1. Research Method

We construct a panel vector autoregression model to explore the dynamic relationship between the tourism industry, technological progress, and ecological economy development, since the PVAR model allows all variables to be endogenous. We collect data from 30 provinces in mainland China (excluding Tibet) from 2007–2019. The research model is set up in this paper as follows.

(1) $$Y_{it}=\alpha_{0}+\overset{n}{\underset{j=1}{\sum }}\alpha_{j}Y_{i,t-j}+\beta_{i}+\gamma_{i}+\varepsilon_{it}\;\left(i,j=1,2,3,\ldots \ldots ,n\right)$$

*Y_it_* = (lntour, lntin, lneco) is a three-dimensional column vector, lntour denotes the tourism industry, lntin denotes the level of technological progress, lneco denotes ecological economy development, and ln denotes the variables taken as logarithms; *α*_0_ is the intercept term; *j* is the lag order; *α_j_* is the parameter matrix with lag order *j*; *β_i_* is the individual fixed effect; *γ_i_* is the individual time-point effect; *ε_it_* is the random disturbance term.

#### 4.1.2. Research Scheme

**Figure 2:**
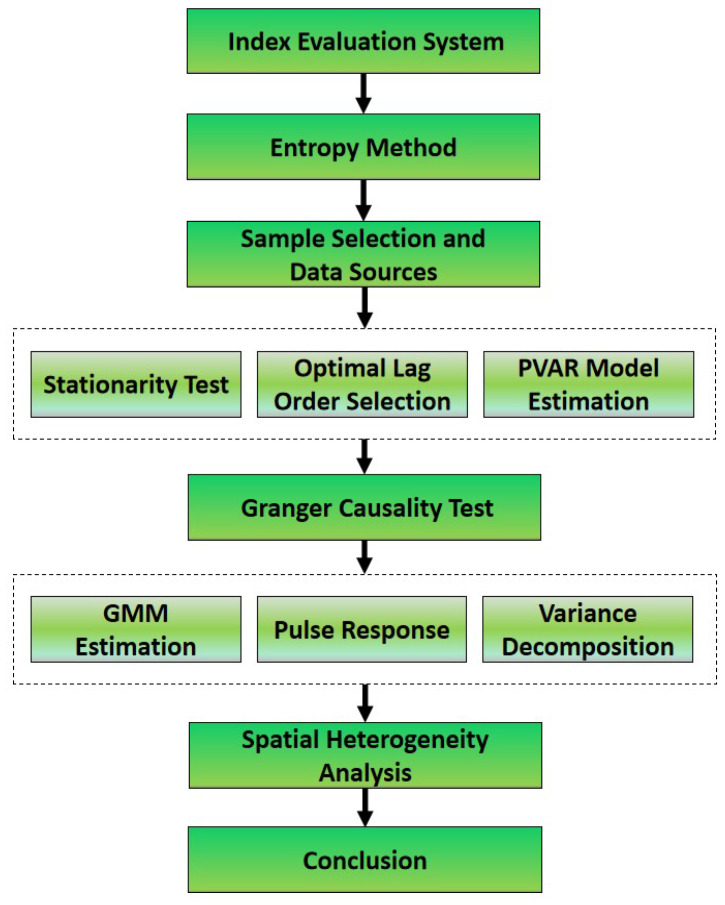
Research scheme of the research process (source: made by authors).

First of all, we elucidate the index indicator of each variable, including the tourism industry, technological progress, and ecological economy development, and analyze the impact mechanism among the three variables. Then, the entropy method is used for all the comprehensive indicators and determines the weight of each sub-index and the final comprehensive indicator score. We also concretely illustrate sample selection and data sources. After that, we perform a stationarity test, determine the optimal lag order, and conduct the PVAR model estimation. In addition, we perform the Granger causality test before we complete the GMM estimation results, the pulse response, and the variance decomposition. Moreover, we perform a spatial heterogeneity analysis to make research conclusions more externally valid and to have higher practical value. Finally, we summarize based on the above analysis and results. The research scheme is shown in Figure 2.

### 4.2. Sample Selection

This paper involves three variables: tourism industry [88,89,90], technological progress [91,92], and ecological economy development [71]. A single indicator can often only focus on one aspect of the variable, which cannot summarize the content represented by the variable as a whole. Thus, based on previous studies, this paper uses comprehensive indicators for all three variables to characterize their connotations. The entropy method is used for all the comprehensive indicators and determines the weight of each sub-index and the final comprehensive indicator score. The main calculation process of the entropy method is as follows.

To determine the sub-index attributes, assign positive and negative indicators, respectively, and standardize the positive and negative indicators, the following formula is used:

(2) $$\left\{\begin{array}{c} x_{ij}=\frac{x_{ij}-\min\left(x_{1j},x_{2j},\ldots x_{nj}\right)}{\max\left(x_{1j},x_{2j},\ldots x_{nj}\right)-\min\left(x_{1j},x_{2j},\ldots x_{nj}\right)}\;i,j=1,2,3,\ldots \ldots ,n\\ x_{ij}=\frac{\max\left(x_{1j},x_{2j},\ldots x_{nj}\right)-x_{ij}}{\max\left(x_{1j},x_{2j},\ldots x_{nj}\right)-\min\left(x_{1j},x_{2j},\ldots x_{nj}\right)}\;i,j=1,2,3,\ldots \ldots ,n \end{array}\right.$$

To calculate the indicator weight, the formula is as follows:

(3) $$p_{ij}=\frac{x_{ij}}{\sum_{i=1}^{n}x_{ij}}\;\left(i,j=1,2,3,\ldots \ldots ,n\right)$$

To calculate the indicator information entropy, the formula is as follows:

(4) $$e_{j}=-\frac{1}{\ln\left(n\right)}*\sum_{i=1}^{n}p_{ij}\ln\left(p_{ij}\right)\;\left(i,j=1,2,3,\ldots \ldots ,n\right)$$

where n represents sample size.

To calculate the indicator weight, the formula is as follows:

(5) $$W_{j}=\frac{1-e_{j}}{\sum_{j=1}^{n}\left(1-e_{j}\right)}\;\left(j=1,2,3,\ldots \ldots ,n\right)$$

To calculate the overall score, the formula is as follows:

(6) $$S_{i}=\overset{n}{\underset{i=1}{\sum }}W_{j}*p_{ij}\;\left(i=1,2,3,\ldots \ldots ,n\right)$$

#### 4.2.1. Tourism Industry (lntour)

The measurement of the tourism industry is based on current studies. An index evaluation system is established from three aspects: industrial base, industrial input, and industrial output. Industrial base indicators include the existing tourism resource base, i.e., the number of scenic spots with AAA ratings, the supply capacity of tourism enterprises including the number of travel agencies, the number of catering enterprises, and the number of accommodation enterprises. The industry input indicators are divided into labor input, i.e., the number of travel agency employees, the number of catering employees, and the number of accommodation employees. The industrial output consists of the total number of tourists of domestic tourism and inbound tourism, and the total revenue of domestic tourism and inbound tourism. The specific index decomposition and weighting are shown in Table 1.

#### 4.2.2. Technological Progress (lntin)

The measurement of technological progress indicators mainly focuses on two aspects, namely inputs and outputs of technological progress, with the full-time equivalent of research and experimental development (R&D) personnel as inputs and the number of patent applications received as the output situation. The specific indicators and their weights are shown in Table 2.

#### 4.2.3. Ecological Economy Development (lneco)

Existing studies have not yet reached a consensus about the measurement of ecological economy development. This paper measures ecological economy development from five dimensions: innovation, coordination, green, openness, and sharing. In addition, it uses the entropy method to assign values to each variable. The specific indicator and the weights assigned are shown in Table 3.

### 4.3. Data Sources

The data on indicators related to the tourism industry development are obtained from the China Tourism Statistical Yearbook 2008–2020 [93], the official websites of the National Bureau of Statistics, and provincial and municipal statistical bureaus. The data of indicators related to technological progress are obtained from the China Science and Technology Statistical Yearbook 2007–2020 [94]. In addition, the data on indicators related to ecological economy development are obtained from the China Statistical Yearbook 2008–2020 [95], the China Environment Statistical Yearbook 2008–2020 [96], the China Energy Statistics Yearbook 2008–2020 [97], the China Science and Technology Statistics Yearbook 2008–2020 [94], and the statistical bulletin of national economic and social development of each province from 2008 to 2020.

## 5. Data Analysis

### 5.1. Stationarity Test and Optimal Lag Order Selection

Although the panel data mitigates the non-stationarity of the data to some extent, the individual variables may still have trend problems and intercept problems that cause pseudo-regression phenomena. To ensure the robustness of the research results, this paper uses three types of unit root tests using the LLC test, ADF test, and PP test for the variables including the tourism industry (lntour), technological progress (lntin), and ecological economy development (lneco). The specific results are shown in Table 4. All three variables passed the unit root test at the 1% significance level, so all three variables are smooth.

Before we conduct the panel vector autoregression (PVAR) model estimation, the optimal lag order of the model should be determined to ensure the validity of the estimated parameters.

In this paper, we use the PVAR2 program package of STATA13.0. to select the optimal lag order by AIC, BIC, and HQIC in three ways, and the specific results are shown in Table 5. As can be seen from the table, under the three detection criteria, the first-order lag order is optimal. Therefore, the PVAR model selection of one-phase lag is the most appropriate. After we determine the optimal lag order of the PVAR model, we can move forward to perform the next test, which can help determine whether the causal relationship depicted by the regression equation is spurious regression or not.

### 5.2. Co-Integration Test

Given the stationarity test, the Pedroni co-integration test was used to examine the long-term equilibrium relationship among the variables. The results of the Pedroni co-integration test are shown in Table 6, rejecting that there is no co-integration relationship among the variables at the 1% significance level. As a consequence, there is a long-term stable equilibrium relationship among the tourism industry, technological progress, and ecological economy development.

As we can see from Table 6, when *p* is zero, the Phillips–Perron t and the augmented Dickey–Fuller t are both negative. However, the modified Phillips–Perron t is positive. The co-integration test can examine whether there is a long-term stable equilibrium relationship among these three variables. However, we still need to conduct a Granger causality test to further examine whether there is a short-term dynamic impact and relationship among them.

### 5.3. Granger Causality Test

To further investigate the short-term dynamic impact effect and logical relationship among the tourism industry, technological progress, and ecological economy development, the Granger causality test is conducted for each variable in this paper, and the results are shown in Table 7.

As can be seen from the table, the tourism industry is the Granger cause of technological progress and economic ecological economy development at the significance level of 10%. The joint action of the tourism industry and ecological economy development is the Granger cause of technological progress. The joint action of the tourism industry and technological progress is the Granger cause of ecological economy development at the significance level of 5%. Therefore, the tourism industry can predict technological progress and ecological economy development to some extent. The joint action of the tourism industry and ecological economy development is crucial in predicting technological progress. The joint action of the tourism industry and technological progress is also valuable in predicting ecological economy development. However, the correlation and the specific predictive relationship among the three variables have to be further explored by tools such as GMM estimation, since GMM estimation is consistent all the time and allows heteroskedasticity and correlation in the model specification.

### 5.4. GMM Estimation Results Analysis

After determining the optimal lag order, this paper uses STATA16 software to perform GMM estimation on the PVAR model constructed by the tourism industry, technological progress, and ecological economy development. The specific estimation results are shown in Table 8. From the table, we can conclude that, firstly, the influence coefficients of the tourism industry, technological progress, and ecological economy development themselves with one lag period are positive and pass the 1% significance level, indicating that all three variables characterize inertial development and self-reinforcing mechanism in development.

Secondly, when the tourism industry is the explained variable, the influence coefficient of technological progress on the tourism industry with one lag period is positive, but it does not pass the significance level, indicating that the promotion effect of technological progress on the tourism industry is not strong in the short term. That is to say, due to technological progress such as big data, virtual reality, intelligent tourism, etc., empowering the tourism industry and promoting its development needs a certain incubation period. The influence coefficient of ecological economy development on the tourism industry with one lag period is negative, but it does not pass the significance test, indicating that there is an inhibitory impact of ecological economy development on the tourism industry in the short term. Hence, this inhibitory effect is weak, since, under the requirement of ecological economy development, policies and regulations as well as development appraisal systems related to the tourism industry need to be reshaped and regulated. The flow of factors and resource allocation of the tourism industry need to be adjusted and optimized, which, to a certain extent, will shock the tourism industry’s wild and sloppy development, resulting in the inhibition of the tourism industry in the short term.

Third, when technological progress is the explained variable, the influence coefficient of the tourism industry with one lag period on technological progress is positive and passes the 5% significance level, indicating that, in the short term, the tourism industry can promote technological progress, which is due to the pull effect and spillover effect generated by the strong correlation between the tourism industry and the primary, secondary, and tertiary industries. For example, technological progress provides the tourism industry with new business models and product patterns that offer tourists a more affluent tourism experience. Certainly, providing such experience greatly relies on emerging tourism technology and equipment, which stimulates the supply of manufacturing, equipment and appliances, and other industries associated with the primary industry, thus promoting innovation, R&D, and technological progress in these industries. The influence coefficient of ecological economy development on technological progress in the lagged period is positive. However, it does not pass the significance test, indicating that the promotion impact of ecological economy development on technological progress is not strong in the short term, and there is a certain lag effect of the production factors accumulated by ecological economy development to enhance technological progress.

Fourth, with ecological economy development as the explained variable, the influence coefficient of the tourism industry on ecological economy development is positive and passes the 5% significance test, indicating that the tourism industry can promote ecological economy development in the short term. The reason is that the tourism industry involves the primary, secondary, and tertiary industries, which have direct and indirect promotion effects, induced effects, and multiplier effects on ecological economy development. Apart from that, the tourism industry is regarded as a smokeless industry, whose industrial development is green and low-carbon, in line with the new development concept and evaluation system for ecological economy development. The influence coefficient of technological progress on ecological economy development is not significant, indicating that technological progress cannot play a role in promoting ecological economy development in the short term, since technological progress has a certain lag effect in generating results and then transforming them into actual benefits. At the same time, the process from the results generated by technological progress to the landing and transformation is likely to be affected by such issues as effective demand not being met, inadequate management system, immature technical system, and poor implementation.

To summarize, though the correlation and the specific predictive relationship among the tourism industry, technological progress, and ecological economy development has been found quite clearly, the GMM estimation is a kind of static analysis, which could not fully meet our needs to more deeply explore the dynamic interaction among them. Therefore, more tools can be applied to further explore the relationship between the tourism industry, technological progress, and ecological economy development.

### 5.5. Impulse Response Analysis

**Figure 3:**
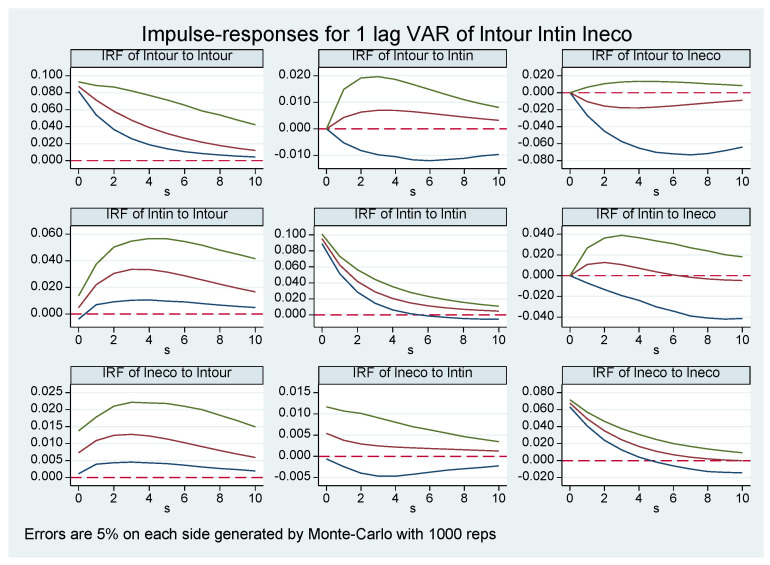
Impulse responses of the tourism industry, technological progress, and ecological economy development of China. The red lines represent estimated impulse response value. The area between the green lines and blue lines is the confidence interval of 5% significance.

The GMM estimation result is a static analysis of the correlation between variables. To further portray the specific dynamic interaction process and response effect between the tourism industry, technological progress, and ecological economy development, the impulse response within a 95% confidence interval and 10-period lag is obtained based on conducting Monte Carlo 1000 simulations (Figure 3). The impulse response refers to the impact on itself and other variables when the random disturbance term is subjected to a standard deviation shock, which can visually reflect the dynamic time-lagged interaction relationship among the variables. From the impulse response diagram, the following can be seen. (1) Each variable responds positively to the shock from itself and reaches its peak in the current period, and then this response gradually declines until it disappears. (2) When the tourism industry is subjected to one standard deviation shock of technological progress, the current period response is zero. In the long run, the tourism industry shows a positive response trend of increasing and then decreasing. It reaches the maximum in the fourth period and finally tends to zero, indicating that the impact of technological progress on the tourism industry development is hysteretic and cumulative, but this impact will gradually weaken. When the tourism industry is subjected to ecological economy development as a standard deviation shock, the current period response is zero, but in the long run, the impact shows a slightly negative response, indicating that the pursuit of ecological economy development will cause a certain inhibitory effect on the tourism industry to a certain extent, perhaps because of the existence of the “crowding out effect”. It also reminds us that we have to use appropriate ways to adjust the tourism industry so it can both ensure its own development and accelerate ecological economy development. (3) When technological progress is subjected to standard deviation shock of the tourism industry, in the long term, it responds positively, showing an “inverted U-shaped” response trend. It reaches a maximum in the third period, so the impact of the tourism industry on technological progress also has a short-term positive cumulative effect. When technological progress is subjected to one standard deviation shock of ecological economy development, the current period response is zero. A positive response is shown in the first six periods, so ecological economy development can promote technological progress in the long run. (4) When ecological economy development confronts the standard deviation shock of the tourism industry and technological progress, it responds positively in our research period, reaches the maximum in the third period, and then gradually shows a decreasing trend. The impact of technological progress on ecological economy development reaches the maximum in the current period and then shows a decreasing trend, both of which can suggest that the tourism industry and technological progress can promote ecological economy development in the long term.

### 5.6. Variance Decomposition

Based on impulse response analysis, we measure the contribution proportion of each variable shock to the fluctuation of endogenous variables by using variance decomposition to further verify the influence degree among variables.

The results of the variance decomposition are shown in Table 9. We can draw a conclusion that the contribution rate of the three variables of the tourism industry (lntour), technological progress (lntin), and ecological economy development (lneco) to themselves is much greater than to the other two variables, which suggests that these three variables have a strong self-enhancement mechanism. Specifically speaking, in period 1, the contribution rate of the tourism industry to itself is 100%, maintaining over 90% in the whole research period. The contribution rate of technological progress to itself is 99.7% in period 1, and the contribution rate decreases gradually over time. However, it still reaches more than 60% in period 10. The contribution rate of ecological economic development to itself is 98.2% in period 1 and is more than 89% in the whole research period.

After investigating the variance decomposition of the tourism industry, technological progress, and ecological economy development, the contribution rate of a structural shock to each endogenous variable is apparent. To further deepen our research results, we develop a spatial heterogeneity analysis, which can visually and directly display the difference between regions in terms of these three variables.

### 5.7. Spatial Heterogeneity Analysis

**Figure 4:**
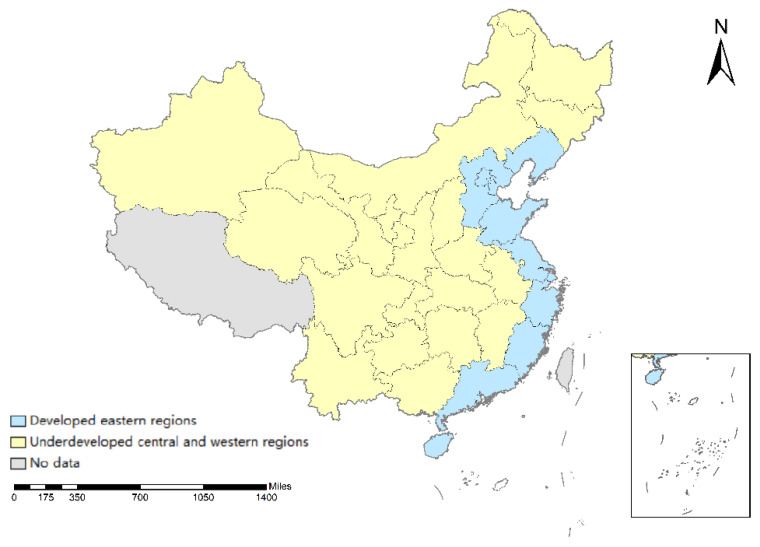
Regional division map of China (source: made by authors).

**Figure 5:**
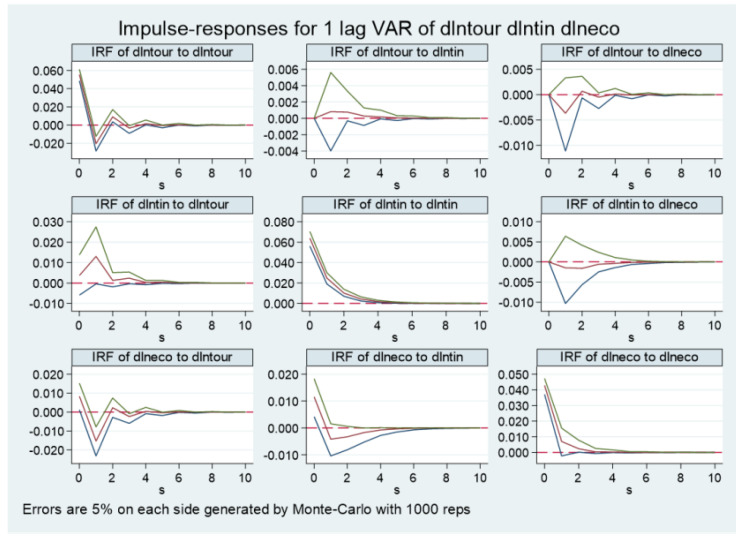
Impulse response analysis of developed eastern regions in China. The red lines represent estimated impulse response value. The area between the green lines and blue lines is the confidence interval of 5% significance.

**Figure 6:**
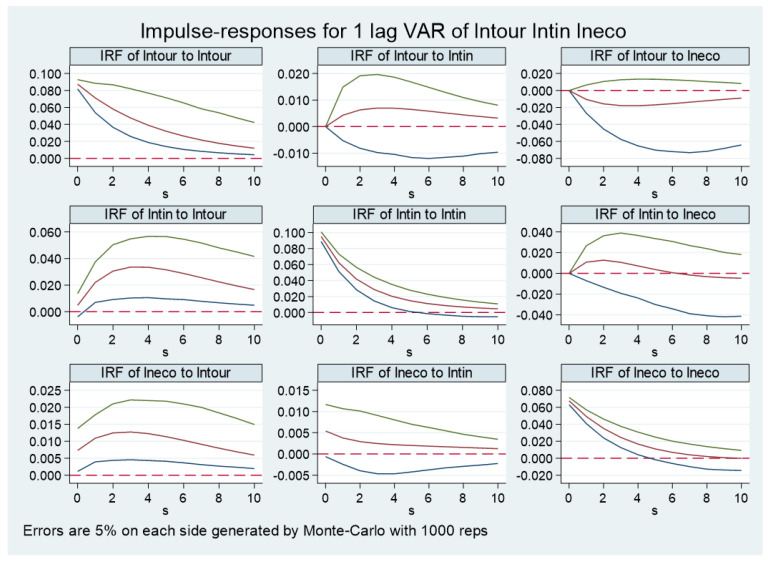
Impulse response analysis of less developed regions in the central and western regions in China. The red lines represent estimated impulse response value. The area between the green lines and blue lines is the confidence interval of 5% significance.

We also conduct a spatial heterogeneity analysis on the relationship among the tourism industry, technological progress, and ecological economy development. The research divides China’s provinces into two categories for analysis and discussion, classifying the eastern coastal provinces of mainland China into the group of developed eastern regions, and the remaining vast central and western provinces into the group of underdeveloped central and western regions. The blue part in the map of China represents the developed eastern regions, while the yellow part stands for the underdeveloped regions (shown in Figure 4). We do not take the grey district into account since we could not gain access to the proper and accurate data. From Figure 5 and Figure 6, it can be seen that, in terms of the impact of the tourism industry on the ecological economy development, the developed eastern regions of China in the current period experience the largest impact, which turns to negative impact, and then back to positive impact, displaying the overall “U-shaped” trend. In the long run, the impact of the tourism industry in the developed eastern region on ecological economy development is positive. Less developed regions in the central and western regions of China experience the greatest impact in the first period, after that, it tends to converge, showing an overall “inverted U-shaped” trend. The impact of the tourism industry in the central and western regions on the ecological economy development is positive in the long run. In terms of the impact of technological progress on ecological economy development, the developed eastern regions confront the largest impact in the current period, which turns to negative, and then converges, showing an overall “U-shaped” trend. In the long run, the technological progress of the less developed regions in central and western China shows a more obvious positive impact on ecological economy development.

## 6. Conclusions and Implications

### 6.1. Conclusions

Based on panel data of 30 Chinese provinces from 2007–2019, this paper uses the entropy value method to accurately measure the tourism industry, technological progress, and ecological economic development, incorporates the tourism industry, technological progress, and ecological economic development into the analysis framework, then empirically tests whether the tourism industry and technological progress have an impact on ecological economic development through a panel vector autoregressive model, and finally uses variance decomposition and the impulse response approach to test the dynamic mechanism of their mutual influence, which leads to the following conclusions.

Firstly, the tourism industry, technological progress, and ecological economy development demonstrate inertial development and a self-reinforcement mechanism in both the short and long term. Secondly, from the perspective of the tourism industry, the impact of technological progress on the tourism industry is not significant in the short term, but has a positive propelling effect in the long term. Nevertheless, the impact of ecological economy development on the tourism industry has a certain inhibitory effect in the short or long term, and ecological economy development has a greater long-term impact on the tourism industry compared to technological progress. Thirdly, from the perspective of technological progress, the tourism industry on technological progress has a positive role in promotion both in the short term and long term. The impact of ecological economy development on technological progress is not significant in the short term, but there is a long-term role in promotion. In comparison, the tourism industry has a greater impact on technological progress. Then, from the perspective of ecological economy development, the tourism industry has a positive role in promoting ecological economy development in both the short and long term. The short-term role of technological progress on ecological economy development is not significant, but there is a long-term role in promoting. On the contrary, the tourism industry has a greater impact on ecological economy development. Next, in terms of the impact of the tourism industry on ecological economy development, the developed eastern regions show a “U-shaped” trend of decline followed by a rise, exhibiting a “crowding out effect”, while less developed regions in the central and western regions show a rapid rise followed by a slow decline in the growth rate of positive promotion influence. Finally, in terms of the impact of technological progress on ecological economy development, the eastern regions show a positive impact in the current period, and then rapidly decline to a negative effect, with a “cost effect”, while the less developed regions in the central and western regions show a strong effect in the current period, then gradually show fluctuating decline in a positive influence.

### 6.2. Theoretical Implications

The main marginal contributions of this research are in three aspects. First, this paper puts the tourism industry, technological progress, and ecological economic development in the same research framework and conducts an in-depth study of the impact of the tourism industry and technological progress on ecological economic development using econometric models. However, most previous studies have used qualitative methods to explore the relationship between the tourism industry and ecological economic development [71] or ecological attributes of the tourism industry in the regional economic sector [98,99]. This study uses quantitative analysis methods to enrich quantitative research in the research field of the tourism industry, technological progress, and ecological economic development. Second, a more comprehensive set of indicators is used to measure the three variables under study. Specifically, this paper comprehensively measures the level of the ecological economic development system in five dimensions: green, innovation, coordination, sharing, and openness. In addition, it also uses the entropy method to effectively measure the tourism industry, technological progress, and ecological economic development. The ecological economic development in current research is merely measured by a single dimension or a simple combination of several indicators [100]. On the other hand, the existing literature has portrayed the ecological economy covering only one specific type of industry, such as coastal tourism or hospitality [101,102], without an adequate three-dimensional and multidimensional indicator system to measure the concept of the tourism industry development [103,104]. Finally, a dynamic panel model, GMM test, and impulse response analysis were used to explore the potential link between the tourism industry, technological progress, and ecological economic development and to conduct a regional heterogeneity analysis. The empirical results indicate that the tourism industry and technological progress can effectively promote the development of China’s ecological economy. Existing studies have neglected the important role played by technological progress between the tourism industry and ecological economic development [105]. However, this study takes a step forward in this research area by obtaining more convincing results based on panel data for 30 provinces from 2007 to 2019. In addition, this study reveals the different pulling effects of the tourism industry and technological progress on ecological economic development in China at different development stages and their advantages, providing theoretical support for the tourism industry and technological progress on ecological economic development.

### 6.3. Policy Implications

First, local governments and tourism authorities should actively implement China’s policies of ecological economic development. On the one hand, the tourism industry, one of the strategic pillar industries of China’s national economy, should play an important role in promoting domestic economic development and reviving the current situation of weak consumption caused by the pandemic. It should strengthen the synergistic linkage among regions and establish a synergistic linkage mechanism to enhance cooperation and exchange in many aspects, including macro policy formulation, technological progress, technology promotion, resource allocation, and complementary advantages. It also should promote the synergistic development of the tourism industry, technological progress, and ecological economy in each region. On the other hand, the blueprint of the tourism industry in macro sustainable development should be formed as soon as possible to explore the driving mechanism of the tourism industry for ecological economy development in terms of energy conservation and emission reduction, technological progress, and green technology utilization. Ecological economy should be used as the core evaluation index for sustainable development of tourism destinations. Energy conservation, emission reduction, and low carbon should be included as the main keywords in various tourism development plans, standard specification systems, and performance appraisal systems of national and local governments. Specifically, ecological indicators such as the use of renewable energy should be incorporated into various regulations of the tourism industry such as the star hotel certification system, the evaluation system of national A-grade tourist attractions, and the construction of ecological tourism demonstration zones.

Second, the concept of ecological economic development should be continuously deepened, and national and regional eco-tourism scenic spots and hotels should be selected to make eco-sustainable development of tourism enterprises a regular branding activity. Local governments, tourism sectors, and related administrative departments can also launch various fiscal and financial policies such as taxes, bonds, funds, credit, and insurance to encourage tourism enterprises to transform to sustainable development. In addition, increasing the use of green technologies and energy-saving equipment is one of the fastest and most pivotal ways to achieve sustainable transformation of China’s tourism industry. Specifically, the use of clean energy, the transformation of green technology innovation, and the promotion of intelligent tourism management should be promoted and strengthened in the whole process and all aspects of the tourism industry production and consumption.

Finally, the tourism industry should constantly improve the technological innovation system, actively guide tourism technological enterprises to increase investment in digital technology research and development, and gradually enhance the independent innovation capability of tourism technological enterprises. The tourism industry should speed up the deep integration with other industries and form a virtuous cycle of mutual promotion between technological progress and tourism industry development. Digital technology is an essential support for the tourism industry to accelerate the integration with other industries and technological innovation and is also one of the crucial measures for ecological economy development. Government authorities should increase the use of digital technology in the upgrading of the regional tourism industry, enhancing tourists’ experiences, and improving destination management.

### 6.4. Limitations and Future Prospects

Numerous studies have confirmed that the tourism industry and ecological economy development in many economies around the world, including China, were greatly influenced by the pandemic [106]. In the post-pandemic era, travel restrictions in mainland China have had a significantly negative impact on tourism industry development. This study collected data prior to the pandemic, but it would be more meaningful and interesting to explore the impact of the tourism industry and technological progress on ecological economic development based on a broader panel of data beyond 2020. Thus, future research should pay more attention to it. In addition, we have portrayed the five dimensions of ecological economic development more comprehensively, but the selection of indicators for technological progress is slightly inadequate. Future research can use a wider range of indicators to better represent technological progress or use indicators in terms of green technological innovation to more finely examine the interactive impact mechanism of technological progress on ecological economic development, which are worthwhile subjects for carrying out in-depth research.

## Figures and Tables

**Table 1:** Index evaluation system and weight for the tourism industry.

First Level Indicator	Second Level Indicators	Third Level Indicators	Indicator Weight
Tourism Industry	Industrial Base	number of scenic spots with AAA ratings (+)	0.433
		number of travel agency enterprises above designated size (+)	
		number of catering enterprises above designated size (+)	
		number of accommodation enterprises above designated size (+)	
	Industrial Input	number of travel agency employees (+)	0.339
		number of catering employees (+)	
		number of accommodation employees (+)	
	Industrial Output	total number of domestic tourism and inbound tourism trips (+)	0.228
		total revenue of domestic tourism and inbound tourism (+)	

Note. “+” indicates positive indicator.

**Table 2:** Index evaluation system and weight for technological progress.

First Level Indicator	Second Level Indicators	Third Level Indicators	Indicator Weight
Technological Progress	Input for Technological Progress	the full-time equivalent of research and experimental development (R&D) personnel (+)	0.393
	Output for Technological Progress	number of patent applications received (+)	0.607

Note. “+” indicates positive indicators.

**Table 3:** Index evaluation system and weight for ecological economy development.

First Level Indicator	Second Level Indicators	Third Level Indicators	Indicator Weight
Ecological Economy Development	Innovation	GDP Growth Rate (+)	0.401
		R&D Investment Intensity (+)	
		Investment Efficiency (−)	
		Trading Activity of Technology (+)	
	Coordination	Demand Structure (+)	0.139
		Urban-Rural Structure (+)	
		Industry Structure (+)	
		Government Debt Burden (−)	
	Green	Energy Consumption Elasticity Coefficient (−)	0.031
		Effluent Generated from Industries (−)	
		Exhaust Emission from Industries (−)	
	Openness	Ratio of Dependence on Foreign Trade (+)	0.347
		Proportion of Foreign Investment (+)	
		Marketization Degree (+)	
	Sharing	Proportion of Labor Remuneration (+)	0.083
		Elasticity of Personal Income Growth (+)	
		Urban-Rural Consumption Gap (−)	
		Proportion of People’s Livelihood Fiscal Expenditure (+)	

Note. “+” indicates positive indicators; “−” represents negative indicators.

**Table 4:** Unit root test results.

	lntour	lntin	lneco
LLC Test	−24.445 ***	−27.448 ***	−15.213 ***
ADF Test	427.657 ***	453.832 **	291.012 ***
PP Test	489.167 ***	550.098 ***	325.186 ***

Note. *** means passing the 1% significance test. ** means passing the 5% significance test.

**Table 5:** Test results of optimal lag order selection.

Lag	AIC	BIC	HQIC
1	−7.581 *	−6.003 *	−6.944 *
2	−6.935	−5.019	−6.158
3	−5.647	−3.298	−4.693

Note. * means the optimal lag order under the code.

**Table 6:** Co-integration tests.

Program	Estimation	*p* Value
Modified Phillips–Perron t	6.164	0.00
Phillips–Perron t	−3.117	0.00
Augmented Dickey–Fuller t	−56.711	0.00

**Table 7:** Granger causality test results.

Program	Causality	Chi-Square	Degree of Free	*p* Value
Technological Progress	Tourism industry development is not the cause.	3.606	1	0.058
	Ecological economy development is not the cause.	1.132	1	0.287
	All variables are not the cause.	7.243	2	0.027
Tourism Industry Development	Technological progress is not the cause.	0.846	1	0.358
	Ecological economy development is not the cause.	1.047	1	0.306
	All variables are not the cause.	2.996	2	0.224
Ecological Economy Development	Technological progress is not the cause.	0.003	1	0.959
	Tourism industry development is not the cause.	2.745	1	0.098
	All variables are not the cause.	7.141	2	0.028

**Table 8:** GMM estimation results.

Variables	lntour		lntin		lneco	
	Coefficient	Z Value	Coefficient	Z Value	Coefficient	Z Value
L1.lntour	0.824 ***	6.49	0.204 **	1.90	0.064 **	1.06
L1.lntin	0.054	0.92	0.647 ***	9.47	−0.002	−0.05
L1.lneco	−0.156	−1.02	0.160	1.06	0.727 ***	10.71

Note. *** means passing the 1% significance test; ** means passing the 5% significance test.

**Table 9:** Variance decomposition results.

Period	lntour			lntin			lneco		
	lntour	lntin	lneco	lntour	lntin	lneco	lntour	lntin	lneco
1	1.000	0.000	0.000	0.003	0.997	0.000	0.011	0.006	0.982
2	0.990	0.001	0.009	0.038	0.954	0.009	0.024	0.006	0.970
3	0.975	0.004	0.022	0.088	0.895	0.017	0.038	0.006	0.956
4	0.959	0.006	0.035	0.139	0.840	0.021	0.052	0.006	0.942
5	0.946	0.007	0.047	0.184	0.794	0.022	0.065	0.006	0.928
6	0.946	0.007	0.047	0.220	0.759	0.022	0.076	0.007	0.917
7	0.925	0.010	0.065	0.248	0.732	0.021	0.086	0.007	0.907
8	0.919	0.011	0.071	0.268	0.712	0.020	0.093	0.007	0.900
9	0.913	0.011	0.075	0.283	0.697	0.020	0.098	0.007	0.894
10	0.910	0.012	0.079	0.294	0.686	0.020	0.102	0.007	0.890

Note. Unit is %.

## Data Availability

Not applicable.
